# Methodologies for high efficiency perovskite solar cells

**DOI:** 10.1186/s40580-016-0074-x

**Published:** 2016-06-30

**Authors:** Nam-Gyu Park

**Affiliations:** School of Chemical Engineering and Department of Energy Science, Sungkyunkwan University (SKKU), Suwon, 440-746 Republic of Korea

**Keywords:** Perovskite, Solar cell, Photovoltaics, Lead halide, Organic inorganic hybrid

## Abstract

Since the report on long-term durable solid-state perovskite solar cell in 2012, perovskite solar cells based on lead halide perovskites having organic cations such as methylammonium CH_3_NH_3_PbI_3_ or formamidinium HC(NH_2_)_2_PbI_3_ have received great attention because of superb photovoltaic performance with power conversion efficiency exceeding 22 %. In this review, emergence of perovskite solar cell is briefly introduced. Since understanding fundamentals of light absorbers is directly related to their photovoltaic performance, opto-electronic properties of organo lead halide perovskites are investigated in order to provide insight into design of higher efficiency perovskite solar cells. Since the conversion efficiency of perovskite solar cell is found to depend significantly on perovskite film quality, methodologies for fabricating high quality perovskite films are particularly emphasized, including various solution-processes and vacuum deposition method.

## Introduction

Organic–inorganic metal halide perovskites with chemical formula ABX_3_ (A = CH_3_NH_3_, B = Pb or Sn, X = I, Br or Cl) were discovered in 1978 [1, 2]. MAPbX_3_ (M = CH_3_NH_3_) changes its color from colorless to orange and to black as anion changes from Cl to Br and to I, respectively, due to decrease in band gap energy. Low band gap iodide perovskite is expected to be a potential candidate for solar cell light harvester, however little attention has been paid to such a possibility because of being keen on change in electrical property depending on structural dimensionality reported in 1994 [3]. In 2009, Miyasaka et al. used MAPbI_3_ and MAPbBr_3_ as light harvesters for the first time in dye-sensitized solar cell structure, in which MAPbI_3_ deposited on nanocrystalline TiO_2_ surface demonstrated a power conversion efficiency (PCE) of 3.8 % [4]. To deposit MAPbI_3_ on TiO_2_, MAI and PbI_2_ were dissolved in gamma-butyrolactone (GBL) and the solution was spin-coated, where Miyasaka group prepared 8 wt% coating solution. It was found that the 8 wt% concentration was too low to induce sufficient coverage of TiO_2_ surface with MAPbI_3_. In 2011, Park et al. solved this problem by modulating coating solution concentration from 10 to 40 wt% and found that 40 wt% solution was enough to cover the TiO_2_ surface, leading dark color even at 3–4 μm thick TiO_2_ film and a PCE of 6.5 % [5]. Absorption coefficient of MAPbI_3_ deposited on TiO_2_ film was found to be one order of magnitude higher than the ruthenium-based organometallic dye coded as N719 adsorbed on the same thick TiO_2_ film. Although these two initial works on perovskite solar cells [4, 5] attracted attention, relatively low PCE values and chemical instability of organic–inorganic hybrid perovskite in polar liquid electrolyte due to ionic characteristics were serious obstacle toward further progress of perovskite solar cell.

In 2012, Park et al. demonstrated a long-term durable high efficiency perovskite solar cell for the first time by replacing a liquid electrolytes with a solid hole-transporting material (HTM), which showed a PCE of 9.7 % at submicron thick TiO_2_ film covered with 2 nm-sized nano dot MAPbI_3_ [6]. This solid-state perovskite solar cell confirmed 500 h stability even without encapsulation because nano dot MAPI_3_ was fully wrapped with hydrophobic spiro-MeOTAD HTM. Two month later, Snaith et al. reported solid-state perovskite solar cell with the same HTM but different oxide Al_2_O_3_, which demonstrated a PCE of 10.9 % [7]. Contrary to the MAPbI_3_–TiO_2_ combination, electron injection is not expected from MAPbI_3_ to Al_2_O_3_ since the condition band position of Al_2_O_3_ is higher than that of MAPbI_3_. This implies that perovskite acts differently from the organic dye molecules requiring electron injection process.

**Fig. 1:**
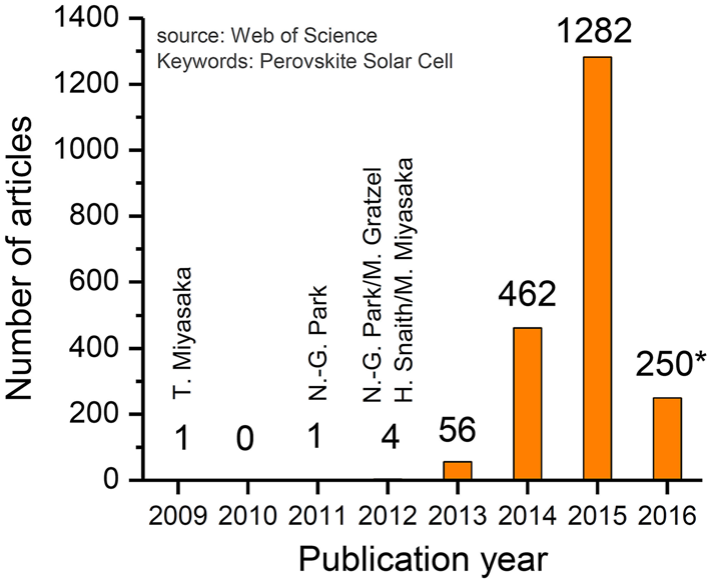
Publications on perovskite solar cells. Data are collected based on Web of Science using keywords “perovskite solar cell”

Reports on solid-state perovskite solar cell with high efficiency and stability have been followed by a surge of interest in perovskite solar cell. Based on Web of Science data (http://ipscience.thomsonreuters.com/product/web-of-science/), around 1300 peer-reviewed research papers on perovskite solar cells were published in 2015 (see Fig. 1), which is almost three time higher than the publications in the previous year. The monthly rate of publication is about 110/month in 2015, which is expected to increase since research papers as many as 250 are already published only within 2 months, as of February 29, 2016. Thanks to the pioneering works on solid-state perovskite solar cell in 2012 [6, 7], a PCE of 22.1 % was achieved in 2016 from the certification institute NREL (www.nrel.gov).

## Review

### Optio-electronic properties of organic lead halide perovskite

Opto-electronic properties of halide perovskites are primarily important in photovoltaics. Absorption coefficient of MAPbI_3_ was first estimated to be 1.5 × 10^4^ cm^−1^ at 550 nm in the form of nanodots deposited in the mesoporous TiO_2_ film [5]. Room temperature absorption coefficients of MAPbI_3_ and MAPbI_3_:Cl films were evaluated by several groups using UV–Vis absorbance data combining with the effect of reflection, spectroscopic ellipsometry considering polarized reflection and photothermal deflection spectroscopy [8–17], which was summarized by Green et al. [18]. All the measured data showed that absorption coefficients range from 2.5 × 10^4^ to 8.9 × 10^4^ cm^−1^ at 620 nm. Among the methods for determining absorption coefficient, ellipsometry may be inaccurate at around band gap transition because high absorption coefficients were shown even below the band edge.

**Fig. 2:**
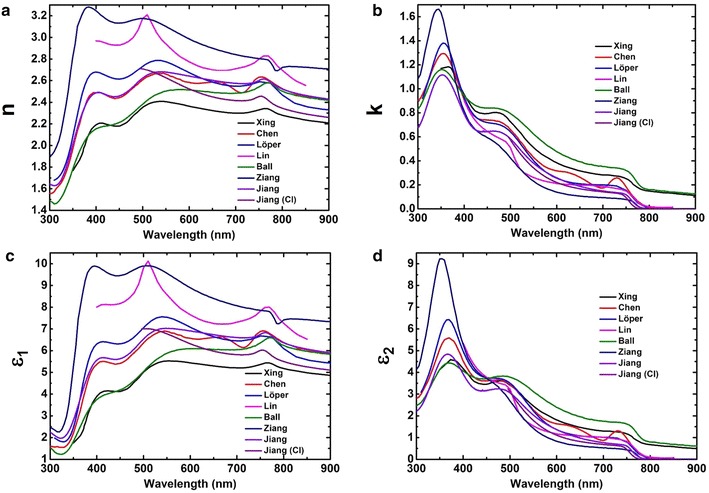
**a** Real (*n*) and **b** imaginary (*k*) part of refractive index and **c** real (*ε*_1_) and **d** imaginary (*ε*_2_) part of dielectric constant of room temperature MAPbI_3_. Reprinted with permission from Ref. [18]

The refractive index is related to how much the speed of light is reduced through a material, compared to the vacuum speed, and the dielectric constant tells us how much the electric field is attenuated in a substance, compared to vacuum. Since light is an electromagnetic wave, two parameters are basically connected each other. Refractive index and dielectric constant for MAPbI_3_ were reported by several groups [8, 10–15], where the real (*n*) and imaginary (*k*) parts of the refractive index are related to those of dielectric constants with the real part (*ε*_1_ = *n* ^2^ − *k* ^2^) and imaginary part (*ε*_2_ = 2*nk*). Imaginary part of refractive index is related to the strength of absorption loss at a particular wavelength (extinction coefficient). Figure 2 shows the real and imaginary parts of refractive index and dielectric constant [18]. A large deviation in the real part of refractive index stems from different layer thickness, morphology, chemical composition, and material anisotropy etc. of the MAPbI_3_ films. The real part of refractive index of MAPbI_3_ ranges between 2.3 and 2.6. Theoretical estimation based on direct band gap energy [19] leads to the refractive index of 2.5 for the bang gap of 1.6 eV of MAPbI_3_, which is well consistent with the measured value. The infrared refractive index of the ABX_3_ halide perovskites can be simply estimated using band gap energy (E_g_) via the simple relationship, *n* ^2^ ≈ 1 + 8.32 eV/E_g_ [18, 19]. The infrared refractive index of MAPbI_3_ (n = 2.5) is lower than that of GaAs (n = 3.3) [20].

Since dielectric constant determines the magnitude of the coulomb interaction between electron–hole pairs and charge carriers as well as any fixed ionic charges in the lattice, high dielectric constants are required for high efficiency solar cell. Usually inorganic materials have higher dielectric constants than organic materials. Dielectric constant for MAPbI_3_ is in the range of 5–7 as can be seen in Fig. 2. Higher value for the relative dielectric constant of MAPbI_3_ was estimated to be about 18 from capacitive measurement [21]. Low effective mass is also required for high efficiency solar cell since effective mass decreases as the carrier becomes more delocalized and its transport becomes more wavelike. Effective mass of electron and hole can be estimated by band structure.

**Fig. 3:**
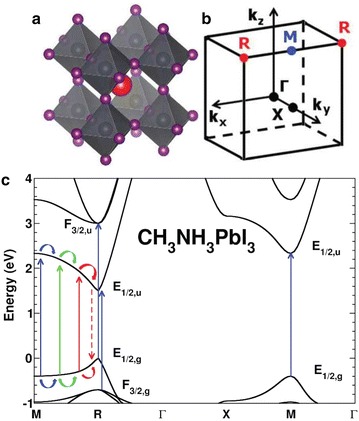
**a** Real-space 3D view of the cubic crystal structure of AMX_3_ (A = Cs, CH_3_NH_3_, M = Pb, Sn, and X = I, Br, Cl) with the Pm3m space group. CH_3_NH_3_^+^ cation (*red ball*) is located at the center of the cube. **b** Reciprocal-space 3D view showing the first BZ of the Pm3m space group. Points of high symmetry in the cubic BZ: Γ denotes the origin of the BZ; X is the center of a square face at the BZ boundary; M is a center of a cube edge; and Rs are vertices of the cube. **c** Electronic band structure for the high-temperature cubic Pm3m phase of MAPbI_3_ with spin–orbit coupling (SOC) at the local density approximation (LDA) level of theory. An upward energy shift of 1.4 eV has been applied to match the experimental bandgap value at R. Irreducible representations obtained from a Pm3m double group analysis are given at R and M points for the electronic states close to the band gap. *Vertical arrows* show various possible optical transitions close to the band gap energy. Optical transitions along the line between the M and R points generate carriers that easily relax toward the R point. Reprinted with permission from 23

MAPbI_3_ was reported to be a direct bandgap material, where the conduction band minimum (CBM) is aligned with the top of the valence band maximum (VBM) at the same effective momentum (k = 0). When the alignment of bandedges in the VBM and the CBM occurs at k = 0, the band structures can be simply obtained using the relation E(k) = ħ^2^k^2^/2m* [22], where ħ (=h/2π), k and m* represent Planck’s constant, effective momentum and effective mass, respectively. Figure 3 displays the band structure and the first Brillouin zones (BZ) for the 3 dimensional cubic MAPbI_3_ [23]. Direct bandgap of MAPbI_3_ is located at the high symmetry point R. In E–k diagram, effective mass can be obtained from second derivatives of E(k) with respect to k, leading to (1/ħ^2^) dE^2^/d^2^k = 2C_1_/ħ^2^ = 1/m_e_* for electron and −2C_2_/ħ^2^ = 1/m_h_* for hole [22]. This indicates that the constants C_1_ and C_2_ in the approximated parabolic curves of CBM for electron and VBM for hole are inversely proportional to effective mass, respectively. As shown in Fig. 3, effective masses of electron and hole are expected to be similar because of similar E–k parabolic feature. In MAPbI_3_ the effective mass of electron and hole was estimated to be 0.23 and 0.29, respectively [24]. The comparable effective mass between electron and hole implies kinetically similar ambipolar characteristics of MAPbI_3_. Although transport of electron and hole is balanced, electron diffusion length (~130 nm) is 1.4 times longer than hole diffusion length (~90 nm) for the solution-processed MAPbI_3_ [25], while hole diffusion length (~800 nm) is 4.6 times longer than electron diffusion length (~180 nm) for FAPbI_3_ [26]. Hall measurement revealed that MAPbI_3_ is close to n-type property but FAPbI_3_ has p-type character [27]. Carrier effective masses along with opto-electronic parameters such as exciton binding energy and dielectric constants are displayed in Table 1 for APbI_3_ perovskites (A = MA and FA) based on a simple two band k.p perturbation theory [28].

**Table 1:** Bandgap (E_g_), exciton binding energy (R*), reduced effective mass (µ), effective dielectric constant (*ε*_eff_) for MAPbI_3_ and FAPbI_3_

Compound	*E*_g_ (meV)	R* (meV)	*μ* (m_e_)	*ε*_eff_	Temperature
FAPbI_3_	1521	10	0.095	11.4	140–160
MAPbI_3−*x*_Cl_*x*_	1600	10	0.105	11.9	190–200
MAPbI_3_	1608	12	0.104	10.9	155–190
FAPbBr_3_	2294	24	0.13	8.6	160–170

Reprinted with permission from Ref. [28]

Charge-carrier mobility plays important role in charge extraction to electrode. Charge-carrier mobility of FAPbI_3_ was estimated to be about 27 cm^2^/Vs as measured by THz photoconductivity transient [29] that is similar to the solution-process MAPbI_3_ [30]. For the mixed-halide perovskite with fromamidinium cation, charge-carrier mobility was found to decrease as bromide content increases from y = 0 to y = 0.5 in FAPb(Br_y_I_1−y_)_3_, significant drop to about 2 cm^2^/Vs at 0.3 < y < 0.5, but recover the mobility up to 14 for the tri-bromide of y = 1 [29], where very low carrier mobility found at 0.3 < y < 0.5 was related to amorphous phase.

Internal PL quantum yield (iQY) is important because it affects directly open-circuit voltage (V_oc_) and photovoltaic performance. The optically implied V_oc_, reflecting the maximum V_oc_ that can be achieved purely based on the intrinsic material quality, assuming no optical losses nor losses caused by nonideal contact architectures, is defined as *q*V_oc_ = E_g_ − T∆S − kT|ln iQY| [31], where *q* is the elementary charge, E_g_ is the band gap, k is the Boltzmann constant, T is the absolute temperature, and S is the entropy. The optically implied V_oc_ was evaluated for MAPbI_3−x_Br_x_ based on the illumination-intensity-dependent maximum iQY of 30 % for 0.1 < x < 1.4 and the entropy of 260 meV in the band gap range of 1.0–1.8 eV. The V_oc_ deficit (E_g_/*q* − V_oc_) was estimated to be about 400 mV at 1 sun up to a band gap of 1.97 eV, which means, for instance, electrical V_oc_ of about 1.2 V can be expected for the band gap of 1.6 eV. The V_oc_ deficit will be further reduced by about 60 mV at optimized carrier injection level [31].

### Methodologies for fabricating high efficiency perovskite solar cells

#### Solution-processed two-step method

Two-step sequential deposition was first proposed by Mitzi et al. [32], where PbI_2_ was deposited on substrate prior to MAI treatment by either vacuum evaporation or spinning coating. The PbI_2_ coated substrate was dipped in MAI solution. Saturated methanol solution of PbI_2_ was used as precursor solution for spin-coating process. The PbI_2_ thin film was immersed in the 2-rpopanol solution containing MAI, which was followed by rinsing with 2-propanol. Dipping time will be crucial to the final product. This two-step method was applied to perovskite solar cell by Gratzel group [33]. Similar procedure was performed, where PbI_2_ layer was formed on the mesoporous TiO_2_ film (average particle size of TiO_2_ was about 20 nm) by spin coating a PbI_2_ solution in *N*,*N*-dimethylformamide (DMF) at 70 °C. The dried PbI_2_ film was dipped in a solution of MAI in 2-propanol for 20 s. It was described that the best efficiency device was obtained from a slight modified method of prewetting of the PbI_2_ film by dipping in 2-propanol for one second prior to being dipped in the MAI solution. A certified PCE of 14.1 % was achieved using the two-step method. As mentioned previously, dipping process, such as dipping time and solution concentration, is crucial to the morphology and opto-electronic property of the final MAPbI_3_ film, associated with the device performance. Two-step spin-coating technology was then proposed to solve the problem occurred by dipping process.

**Fig. 4:**
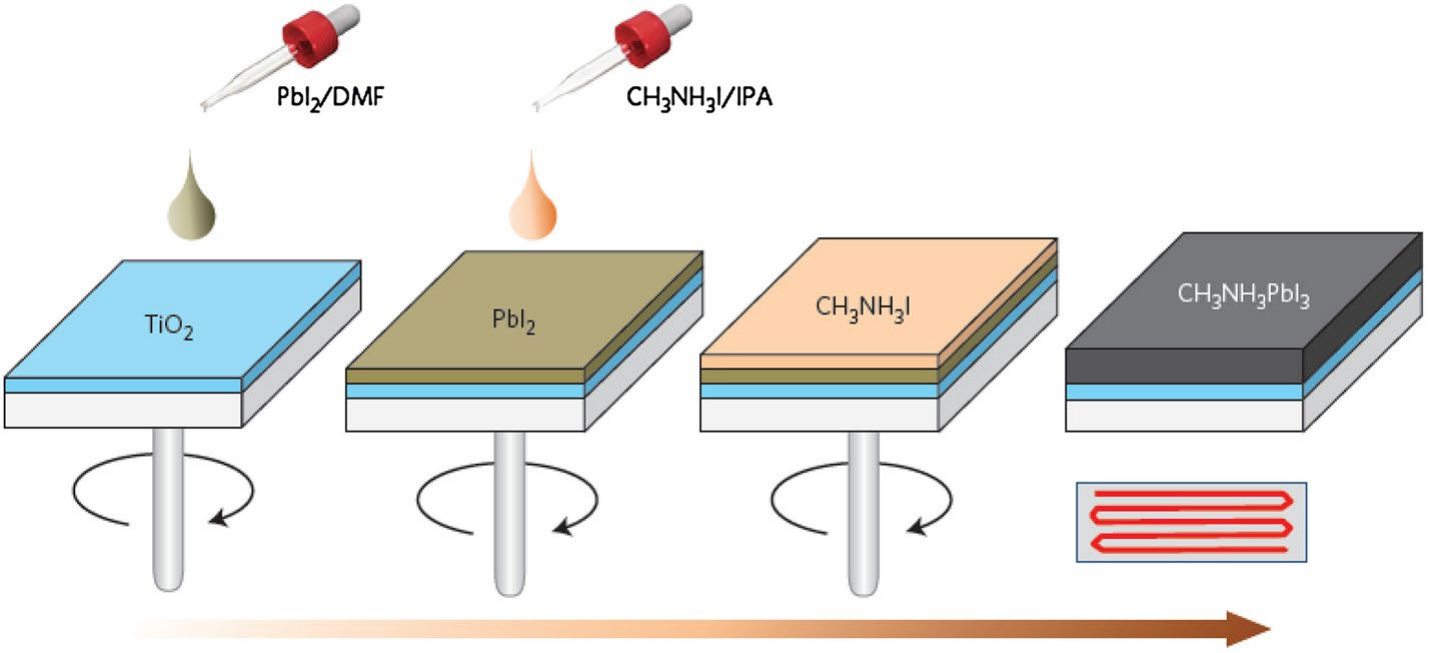
A PbI_2_ solution is first spin-coated onto the mesoporous TiO_2_ film. The PbI_2_-coated film is dried at 40 °C for 3 min and 100 °C for 5 min. A MAI solution in 2-propanol is then loaded onto the dried PbI_2_ film for 20 s and spun. Finally, the film is heated at 100 °C for 5 min. Reprinted with permission from Ref. [34]

**Fig. 5:**
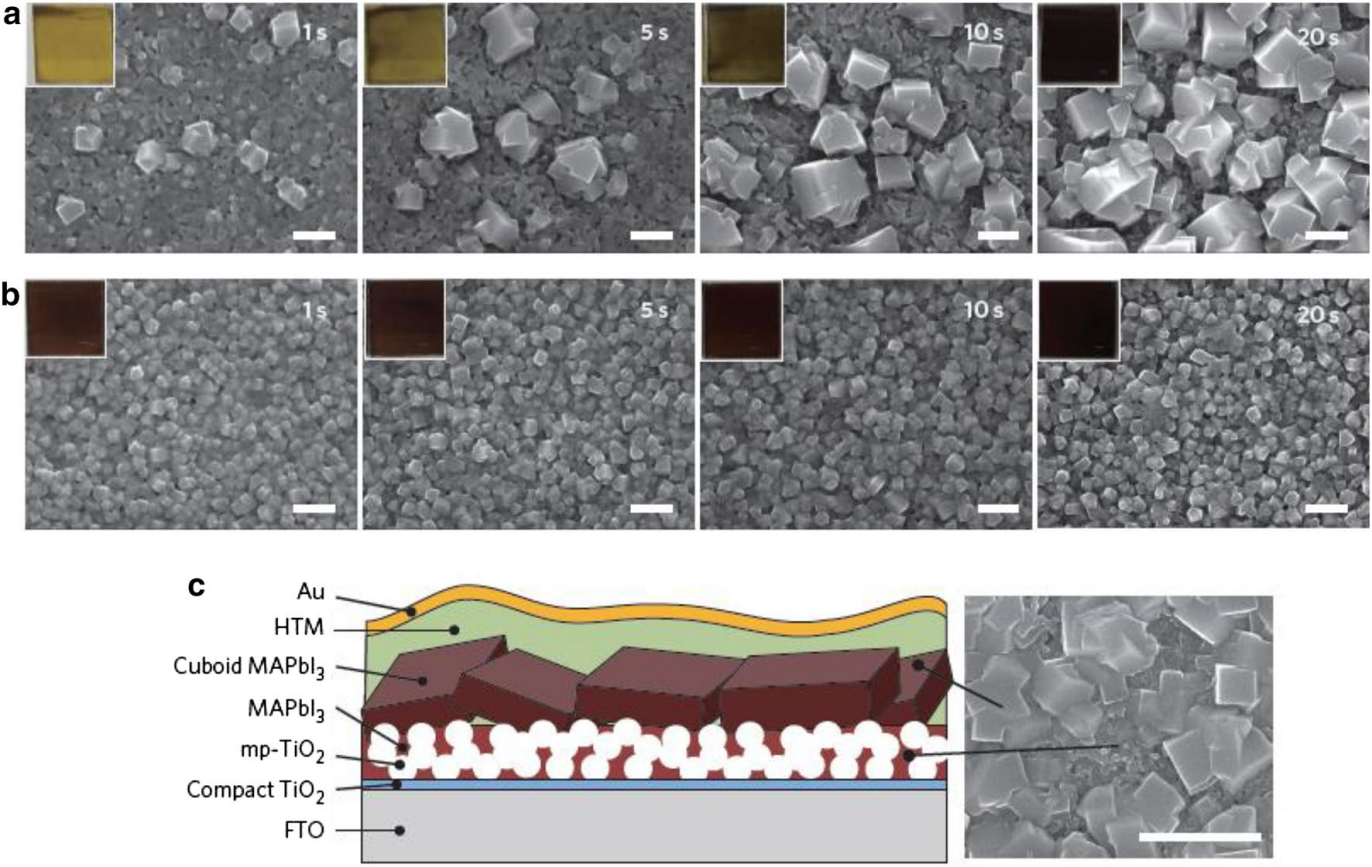
Surface scanning electron microscopy (SEM) images at different loading times for **a** the low concentration of 0.038 M and **b** the high concentration of 0.063 M MAI solutions. Insets show photographs of the samples used for SEM measurement. *Scale bars* 500 nm. **c** Schematic of the perovskite solar cell configuration based on surface and cross-sectional SEM images, where large cuboid MAPbI_3_ was formed on top of the mesoporous TiO_2_ layer. *Scale bar* (SEM image), 1 μm. Reprinted with permission from Ref. [34]

A 2-propanol solution of MAI was spin-coated on the PbI_2_ film, instead of dipping the PbI_2_ film in the MAI solution, which was found to create nanocubic perovskite morphology and its size was significantly dependent on the concentration of MAI solution [34]. In Fig. 4 the two-step spin-coating procedure is depicted, where perovskite crystal size and photovoltaic performance were found to be significantly influenced by the MAI concentration. For instance, low concentration produced large cuboid crystal but high concentration yielded small cuboid MAPbI_3_. Time-dependent crystal growth study revealed that small seed crystals were sparsely grown on the substrate initially for the low MAI concentration, which grew further with time, whereas small seed crystals were fully occupied on the substrate in early stage for the high MAI concentration, inhibiting further growth, as can be seen in Fig. 5. Crystal growth mechanism was explained by thermodynamic Gibbs free energy change [35]. The cuboid size was found to be correlated with concentration of MAI and temperature, where the observed cuboid size depending on the MAI concentration was well fit with the proposed equation [35].

**Fig. 6:**
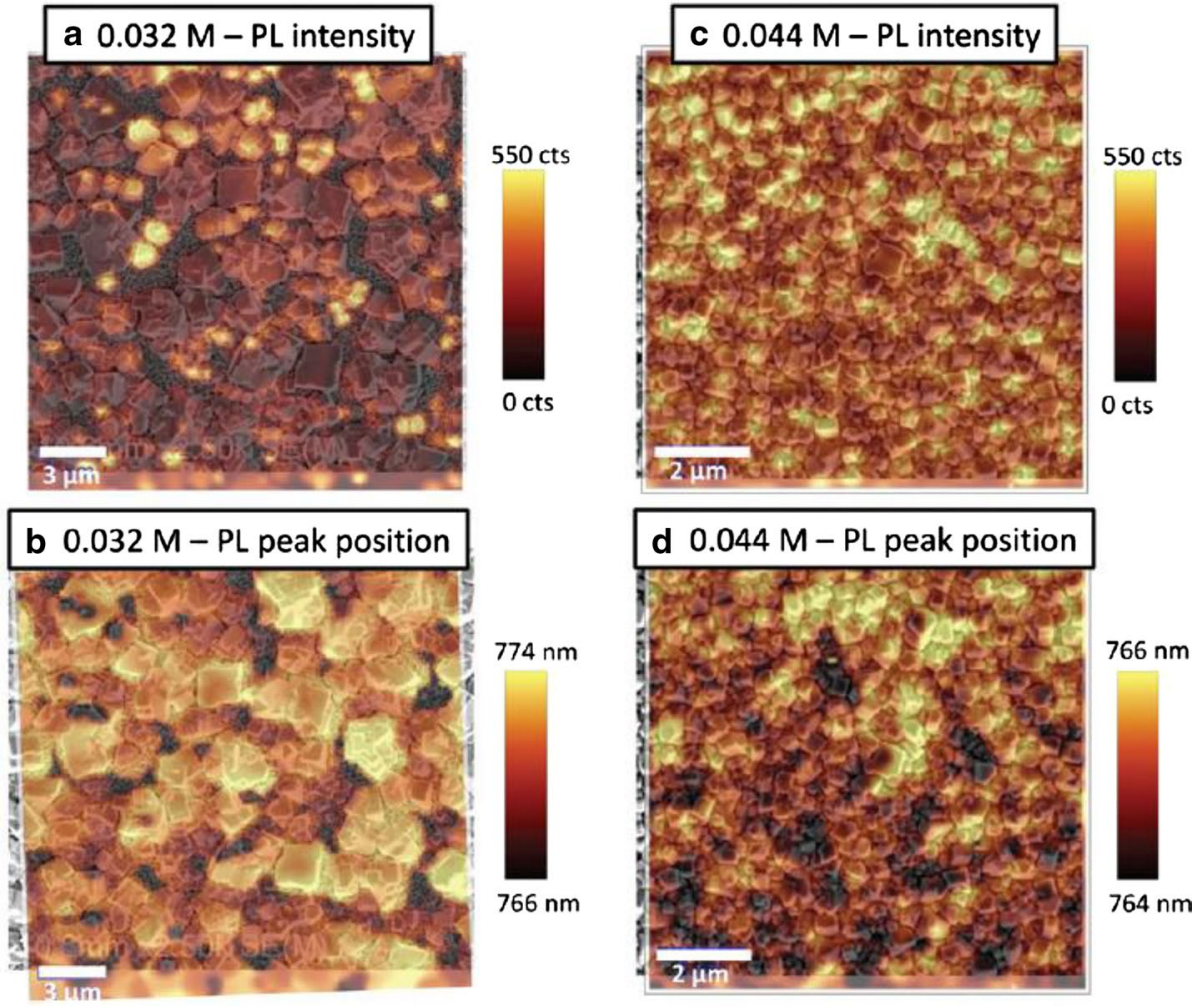
**a**, **c** μ-PL intensity and **b**, **d** spectral position of the PL peak for the layout of TCO/bl-TiO_2_/mp-TiO_2_/MAPbI_3_ with 0.032 M and 0.044 M MAI concentration. High resolution μ-PL imaging overlaid with SEM images acquired on the same spot. Note the different *color bars* in **b**, **d** which is 766–774 nm for the 0.032 M sample and has a much smaller range (764–766 nm) for the 0.044 M sample. The *scale bar* is 3 μm for the 0.032 M film and 2 μm for 0.044 M. Reprinted with permission from Ref. [36]

The small cuboids obtained by high MAI concentration were closely packed on the substrate, but the large cuboids produced gaps between the cuboids. Thus, large cuboid film exhibited better light harvesting efficiency due to enhanced internal light scattering, leading to higher photocurrent density (J_sc_). However, largest cuboid (~800 nm) did not show highest open-circuit voltage (V_oc_) among the studied sizes ranging from ~90 to ~800 nm. Instead, medium size of about 200 nm showed highest V_oc_. According to the photo-CELIV (charge extraction by linear increasing voltage) study, fast charge mobility along with high charge extraction ability was observed for the medium sized MAPbI_3_ cuboid compared to larger or smaller sized ones. Retarding charge mobility may increase the chance of recombination, being responsible for lowering V_oc_ for the largest size. This indicates that the mobility for charge extraction plays important role in managing V_oc_. Since J_sc_ was observed to increase with increasing the MAPbI_3_ size, one can expect higher J_sc_ from the larger size. However, higher J_sc_ could not be obtained from the a few micron sized MAPbI_3_ grown by further decreasing the MAI concentration such as 0.032 M. J_sc_ (12.8 mA/cm^2^) for the MAPbI_3_ film prepared from 0.032 M was only 56 % of that (22.8 mA/cm^2^) for the one prepared from 0.044 M MAI solution [36]. Micro photoluminescence (µ-PL) studies revealed that the 0.032 M cell had a substantially lower radiative recombination than the 0.044 M one, which eventually led to much lower charge collection efficiency. High resolution µ-PL mapping was investigated to compare the PL intensity and spectral position of the PL peak. In Fig. 6, a strong inhomogeneity in both intensity and spectral position of the peak is observed for the perovskite film prepared with the 0.032 M MAI solution, in which largest crystals show the lowest PL intensity along with red-shift of the PL peak associated with reduced bandgap energy. Such a low PL intensity stems from non-radiative recombination that could be related to crystallinity, grain boundary, trap states and surface defects of perovskite film. Therefore, overall performance of perovskite solar cell is strongly influenced by the electrical and radiative properties of perovskite film.

Two-step deposition technique was found to be beneficial to fabrication of perovskite film at relatively high humidity condition. The substrate pre-heating process for PbI_2_ deposition in the two-step spin-coating procedure was found to be crucial to the final MAPbI_3_ morphology and photovoltaic performance, where infiltration of PbI_2_ in the mesoporous TiO_2_ film was better for the heated substrate than for the substrate without heating [37]. PCE increased with increasing the substrate temperature from room temperature to 50 °C and then decreased upon further increasing temperature to 60 °C, exhibiting optimal substrate temperature of around 50 °C. The pre-heating method was found to be not suitable for one-step coating under high relative humidity environment. Humidity effect in fabrication process was examined, where the relative humidity less than 60 % was hard to affect the overall performance [38].

Modified two-step deposition methods were proposed. Vapor treatment of organic solvents such as toluene or chlorobenzene on the PbI_2_ films resulted in better photovoltaic performance because the increased grains size and surface area of the PbI_2_ layer provided better reaction sites for MAI [39]. Interdiffusion method was proposed to fabricate MAPbI_3_ without thermal annealing, where a MAPbI_3_ film formed from the MAI/PbI_2_ bilayer film in air exposure for 30 min at relative humidity of about 30 % demonstrated a comparable performance to the thermally annealed perovskite [40]. In the two-step process, instead of depositing PbI_2_ layer, PbO film was electrochemically deposited on a conductive substrate before reaction with MAI at 150 °C for 30 min. [41]. A possible reaction mechanism was proposed as follows. The MAI is decomposed to CH_3_NH_2_ and HI at the elevated temperature at the initial stage, and the generated HI is reacted with the PbO to form PbI_2_. Finally the PbI_2_ is reacted with MAI to form MAPbI_3_. The equivalent amount of H_2_O generated during the conversion process from PbO to PbI_2_ was argued to have positive effect on the formation of the provskite layer. To prevent volume change in two-step sequential deposition method, a intermediate PbI_2_(DMSO)_x_ was pre-deposited before treatment of organic ammonium halides, which led to a PCE more than 20 % [42].

#### Solution-processed single precursor and anti-solvent method

**Fig. 7:**
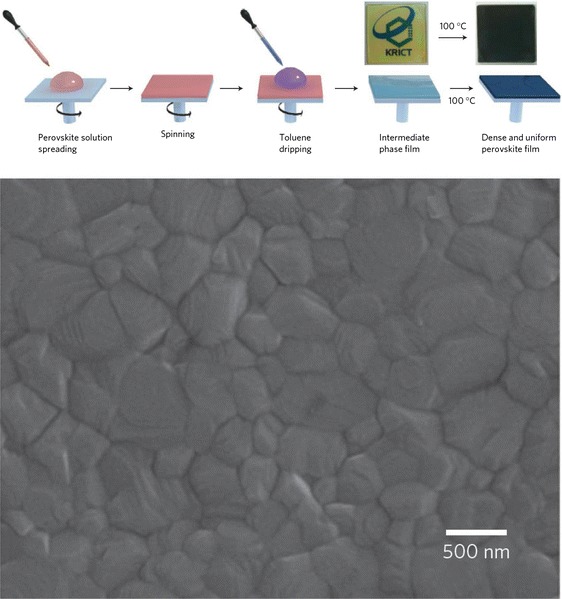
(*Top*) a schematic illustration of solvent engineering procedure and (*bottom*) SEM image of the MAPbI_3_ produced by the solvent (toluene) engineering method. Reprinted with permission from Ref. [44]

One-step method seems to be better in terms of minimizing the processing step. However, photovoltaic performance of device made by a simple one-step method is inferior to two-step results because of significant difference in morphology of the MAPbI_3_ [43]. Thus morphology control is crucial to one-step method. Anti-solvent engineering was proposed to control the crystal growth kinetics [44]. The precursor of MAI and PbI_2_ was dissolved the mixed solution of *N*,*N*-dimethylsulfoxide (DMSO) and GBL, which was spin-coated on the substrate. Anti-solvent such as toluene was dripped while spinning the precursor solution, which led to homogeneous and flat perovskite film with well-developed large grains. Figure 7 illustrates a schematic procedure of anti-solvent treatment for one-step deposition method, along with the morphology of MAPbI_3_ produced by this method. By utilizing the solvent engineering technique, high PCE of 18.4 % was achieved from the solid solution between FAPbI_3_ and MAPbBr_3_ with ratio of 85 %:15 % [45].

**Fig. 8:**
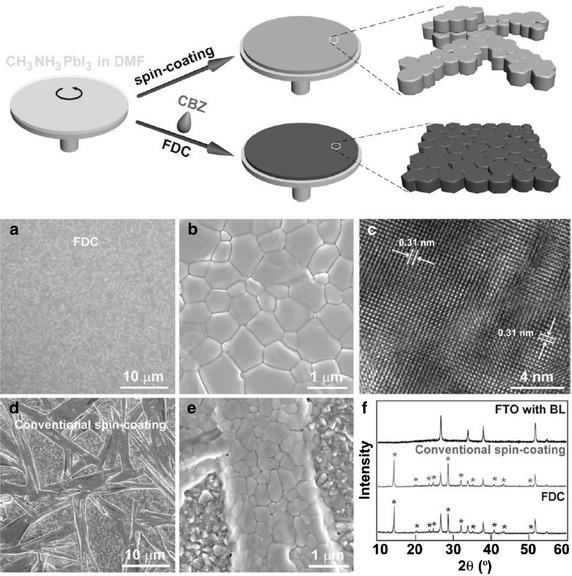
(*Top*) schematic illustration of the FDC process and conventional spin-coating process for fabricating perovskite films. In the FDC process, a second solvent (e.g. chlorobenzene) introduced on top of the wet MAPbI_3_ film during the spin-coating process induces fast crystallization of uniformly sized perovskite grains. **a**, **b** Low- and high-magnification SEM plane-view images and **c** high-resolution TEM image of a MAPbI3 film prepared by FDC with the addition of chlorobenzene. **d**, **e** Low- and high-magnification SEM images of a film prepared by conventional spin-coating without using anti-solvent. **f** XRD patterns for the films for FDC method and conventional spin-coating method. Reprinted with permission from Ref. [46]

Fast deposition-crystallization (FDC) procedure was proposed to produce uniform and flat MAPbI_3_ film [46] to overcome non-uniform MAPbI_3_ film with pinholes that is usually produced by one-step deposition of a DMF solution containing MAI and PbI_2_ because of slow crystallization due to high boiling point of DMF (153 °C). Detailed FDC method is as follows. A DMF solution of MAPbI_3_ was first spin-coated on the bl-TiO_2_ layer. After a specific delay time, a second solvent (anti-solvent) was quickly added to the substrate, where the use of the second solvent is to promote fast nucleation and growth of MAPbI_3_ by reducing the solubility of MAPbI_3_ in the mixed solvent. 12 solvents were tested, including chlorobenzene, benzene, xylene, toluene, methanol, ethanol, ethylene glycol, 2-propanol, chloroform, THF, acetonitrile, and benzonitrile. Figure 8 shows the schematic procedure of FDC using anti-solvent of chlorobenzene and the difference in morphology of MAPbI_3_ between with and without anti-solvent, where large grain approaching micron scale and highly crystalline nature are clearly shown for the perovskite prepared by FDC method.

#### Solution-processed adduct method

**Fig. 9:**
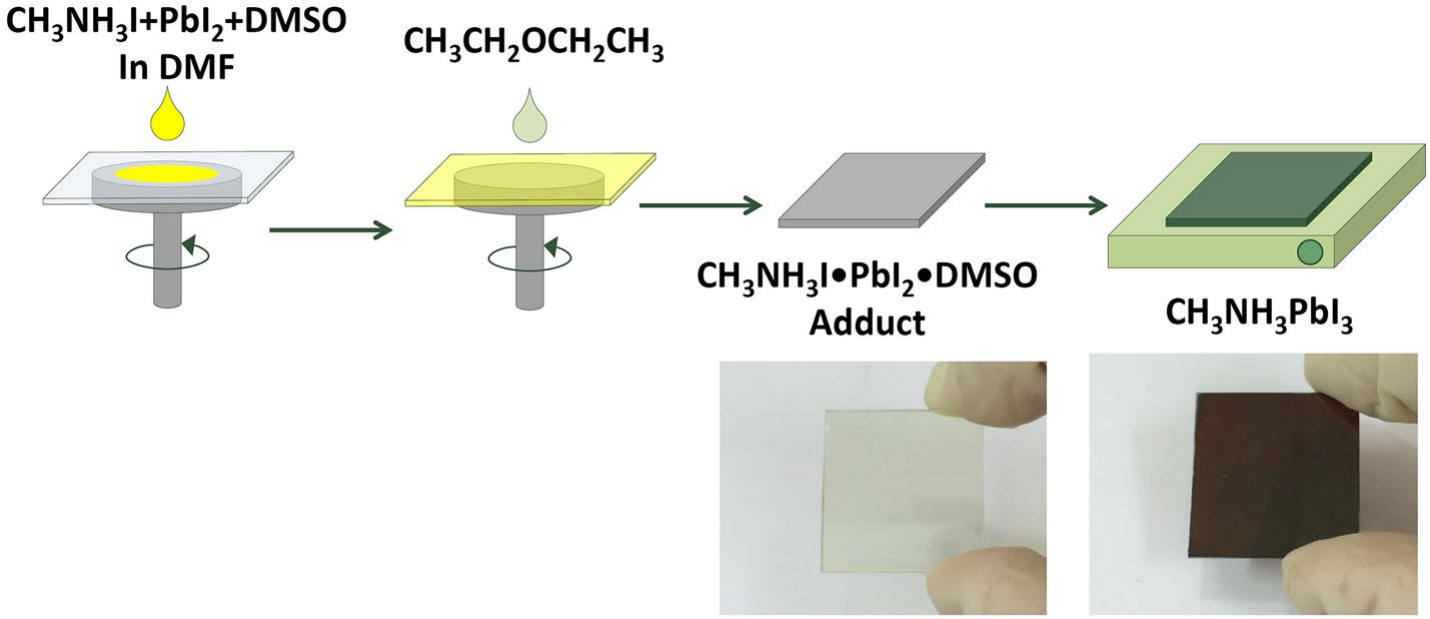
A schematic procedure of the adduct method. The MAI·PbI_2_·DMSO adduct film is transparent, which is converted to *dark brown* MAPbI_3_ upon removal of DMSO from the adduct. Reprinted with permission from Ref. [47]

As one of effective methods, Lewis acid–base adduct approach was proposed to prepare high quality of MAPbI_3_ perovskite film [47]. The interaction between DMSO as a Lewis base and PbI_2_ as a Lewis acid led to a transparent adduct film which was converted to MAPbI_3_ by removing DMSO at mild heat treatment. In Fig. 9, a schematic procedure of the adduct approach is presented. The equimolar mixture of PbI_2_, MAI and DMSO in DMF solvent is spin-coated on a substrate and then diethyl ether is dripped while spinning, which eventually results in a transparent film that is directly indicative of the formation of adduct. The purpose of using diethyl ether is to remove only DMF to form the 1:1:1 adduct film. Thermally removal of DMSO from the adduct film controls kinetically the MAPbI_3_ growth. FTIR is good tool to confirm the adduct formation, where a stretching vibration of S=O was found to shift from 1045 cm^−1^ for DMSO solvent to 1020 cm^−1^ by interacting PbI_2_ with DMSO and to 1015 cm^−1^ for the MAI·PbI_2_·DMSO adduct [47].

The adduct-induced MAPbI_3_ layer showed flat surface with large grains. A device employing adduct-induced MAPbI_3_ demonstrated charge carrier mobility of 3.9 × 10^−3^ cm^2^/Vs (the value was measured by photo-CELIV, which was lower than the value (~30 cm^2^/Vs) obtained by THz method), which was one order of magnitude higher than that (3.2 × 10^−4^ cm^2^/Vs) of MAPbI_3_ prepared by a simple one-step method [48]. Charge extraction characteristics was improved by the adduct method, which may be ascribed to better PL quantum yield. The best PCE of 19.7 % was achieved by using the adduct method.

**Fig. 10:**
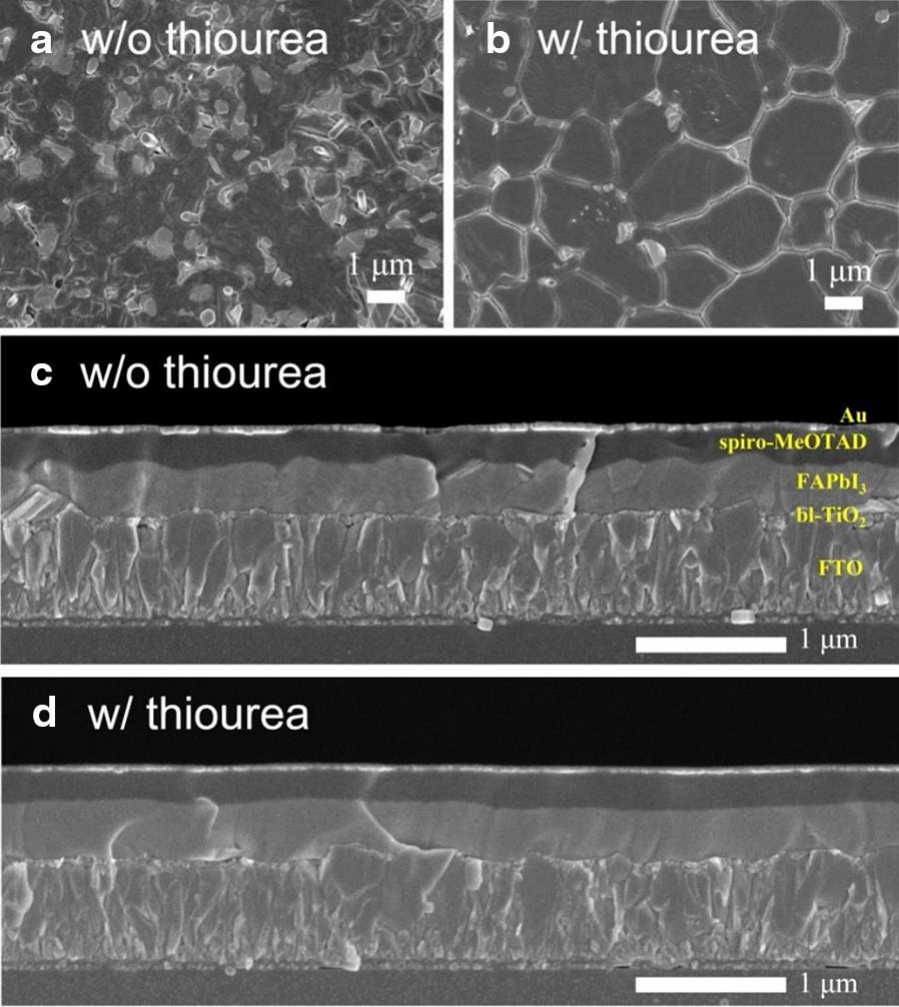
Plane-view and cross-sectional SEM images of FAPbI_3_ perovskite layers formed by adduct approach **a**, **c** without thiourea (only DMSO) and **b**, **d** with thiourea (20 % thiourea and 80 % DMSO). Reprinted with permission from Ref. [49]

Since the adduct approach is generally adapted in the presence of Lewis acid and base if their frontier orbital energies are similar, this adduct approach was applied to fabrication of FAPbI_3_ layer. In this case, selection of Lewis base may affect the final film quality. DMSO was found to an effective Lewis base for preparing high quality MAPbI_3_ film. But, for the FAPbI_3_ case, DMSO may not be good choice because methyl group in DMSO is not matched with HC(NH_2_)_2_ cation in FAPbI_3_. Similarity of functional group is considered, where thiourea is expected to be better because of similarity of functional group between thiourea and FA. Figure 10 shows that introduction of thiourea results in highly uniform FAPbI_3_ film with much larger grains (from 1 to 4 μm) compared to without thiourea case (from 10 to 1 μm) [49], which is likely to correlate with stronger interaction of thiourea in adduct than DMSO, giving kinetically controlled crystal growth. XRD measurement confirmed that the X-ray crystallite size of FAPbI_3_ was significantly enhanced from ca. 50 to ca. 120 nm when 20 % thiourea was added. Addition of thiourea in adduct formation showed higher PCE along with reduced I–V hysteresis compared to the FAPbI_3_ prepared using only DMSO.

#### Vacuum deposition method

**Fig. 11:**
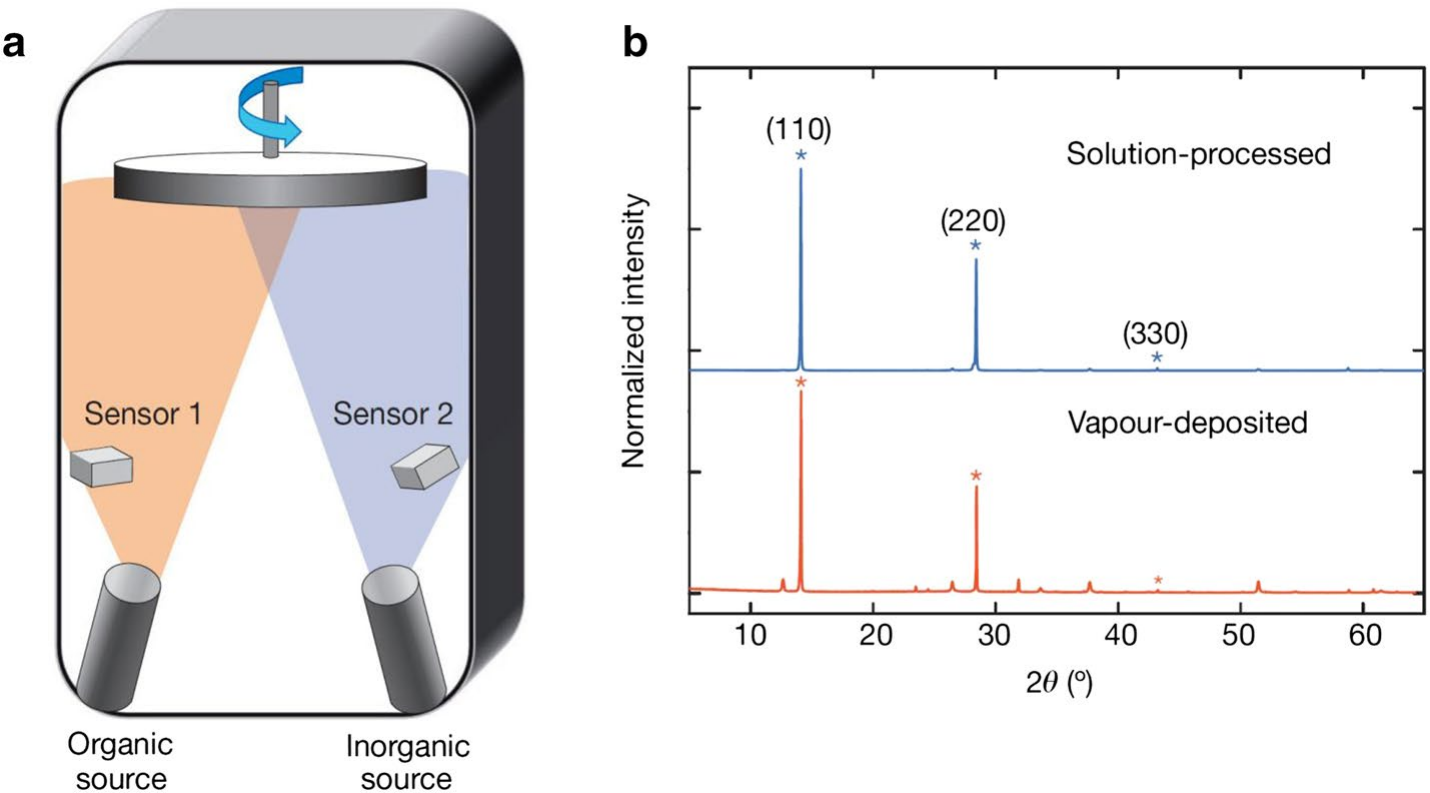
**a** Dual-source thermal evaporation system for depositing MAPbI_3_ using MAI and PbI_2_ (or PbCl_2_). **b** X-ray diffraction patterns of a solution-processed MAPbI_3_ film (*blue*) and vapor deposited one (*red*). Reprinted with permission from Ref. [50]

Since PbI_2_ can be deposited by thermal evaporation [32], organo lead halide film can be prepared by vacuum deposition process. Snaith et al. reported first the vacuum-deposited MAPbI_3_ film and applied it to the planar heterojunction perovskite solar cell [50]. Figure 11 shows the thermal evaporation process using dual sources of PbI_2_ (or PbCl_2_) and MAI and X-ray diffraction pattern of the vapor deposited MAPbI_3_ that is compared with the solution-processed one. In the reference 50, the vapor-process was argued to be better than the solution-process in planar heterojunction layout because the former produced a flat and even surface.

**Fig. 12:**
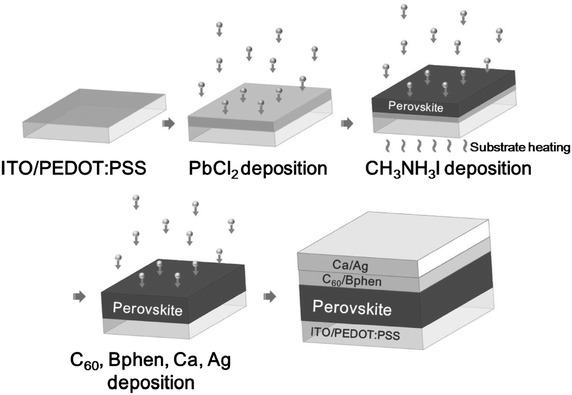
Schematic illustration of perovskite solar cells fabricated by sequential layer-by-layer vacuum deposition. Reprinted with permission from Ref. [51]

A sequential layer-by-layer vapor deposition was proposed similar to two-step solution process, where PbCl_2_ was first deposited by thermal evaporation, which was followed by vapor deposition of MAI [51]. This sequential deposition was developed because of difficulty in monitoring of MAI deposition rate in co-deposition process. Substrate was heated at temperature ranging from 65 to 85 °C, in which photovoltaic performance was found to depend significantly on the substrate temperature. Higher performance was observed from the MAPbI_3_ layer deposited at 75 °C. In Fig. 12, a schematic process for sequential vapor-deposition of perovskite film is illustrated.

Since it is difficult in adjusting stoichiometry in co-deposition vacuum process, stoichiometric control is important. Inductively coupled plasma mass spectrometry (ICP-MS) was used to get the quantitative I/Pb ratio, where omnidirectional MAI evaporation could be controlled using the chamber pressure and incorporated in the film through interaction with the unidirectionally evaporated PbI_2_ [52]. I/Pb was linearly proportional to chamber pressure, from which a chamber pressure of 1.23 × 10^−4^ mbar and a perovskite deposition rate of 0.03 nm/s produced stoichiometric MAPbI_3_. It was noted that UV–Vis spectral feature and PL peak position were almost identical regardless I/Pb ratio, which indicates that UV–Vis and PL are limited to determine the stoichiometry of MAPbI_3_.

#### Combined method

**Fig. 13:**
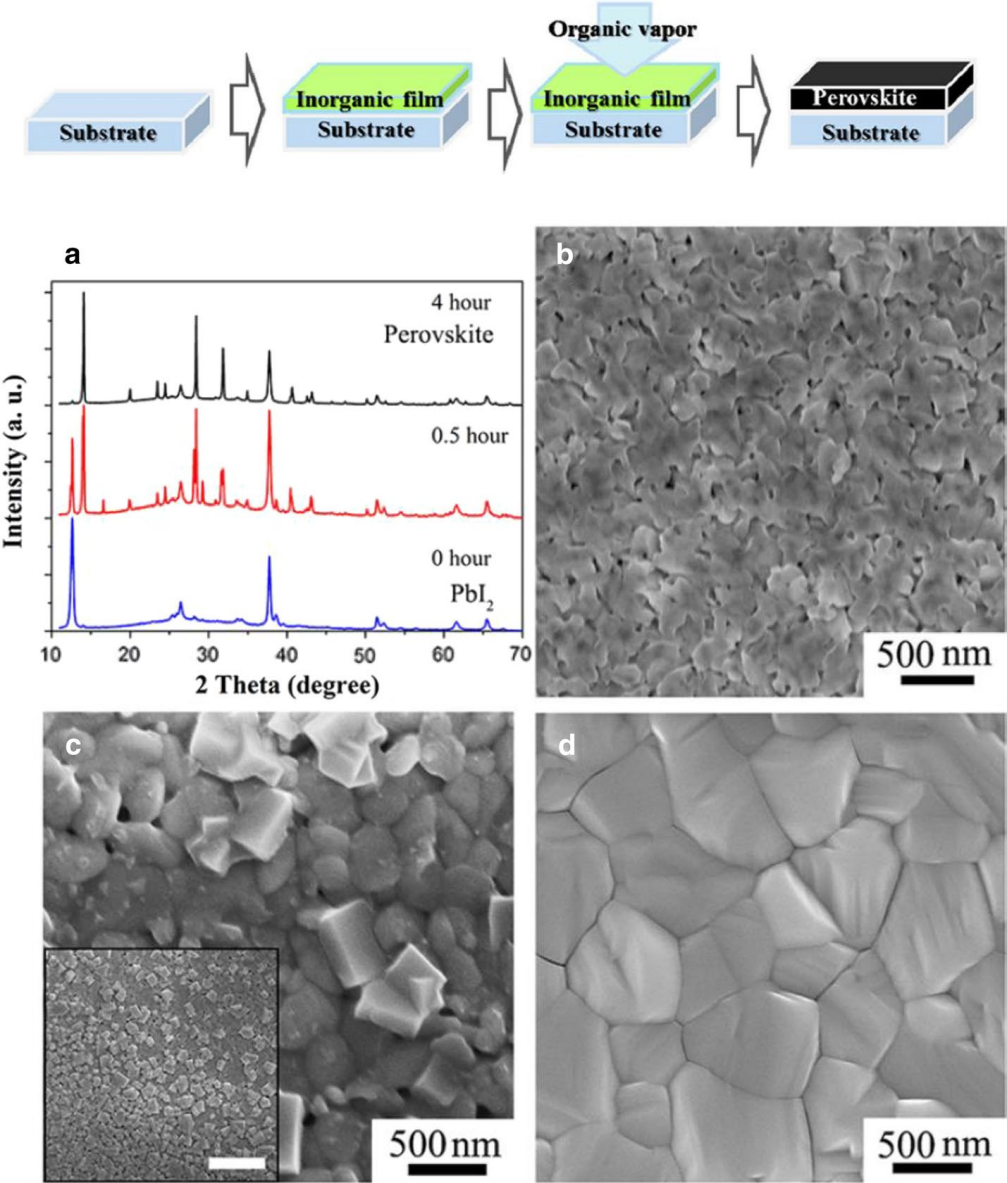
(*Top*) schematic illustrate of the vapor assisted solution process (VASP) and (*bottom*) XRD and SEM images of the perovskite thin film obtained by annealing PbI_2_ film (~200 nm) in the presence of MAI vapor at 150 °C in N_2_ atmosphere. **a** XRD patterns of the film annealed at 0, 0.5, and 4 h, **b**–**d** plane-view SEM images of **b** the initial stage at 0 h, **c** the intermediate stage at 0.5 h (*inset* wider view, *scale bar* 3 μm), and **d** the post stage at 4 h. Reprinted with permission from Ref. [53]

The vapor assisted solution process (VASP) was proposed to fabricate perovskite thin films, where the solution-processed PbI_2_ film was treated with MAI vapor [53]. This method was developed to avoid co-deposition of organic and inorganic species. VASP provided films with well-defined grain structure with grain sizes up to microscale and small surface roughness. The as-deposited PbI_2_ films were annealed in MAI vapor at 150 °C in N_2_ atmosphere for 2–4 h to form the uniform and large grain perovskite films. Figure 13 shows the grain growth of MAPbI_3_ with time, where annealing time of 30 min is not enough for full conversion of PbI_2_ to MAPbI_3_ but annealing for 2 h or longer completes the conversion process. The MAPbI_3_ layer thickness was found to be increased compared to the pristine PbI_2_ layer thickness. It should be noted that ionization energy of MAPbI_3_ film was varied from 5.67 to 6.4 eV depending on preparation methods due to non-stoichiometry of final product, which was found to influence photovoltaic performance [54].

## Summary and outlook

In this review, opto-electronic properties of MAPbI_3_, FAPbI_3_ and perovskites with mixed halide anions were investigated. Refractive index, dielectric constant, effective mass and charge diffusion length are important parameters for light absorbing, charge transporting and collecting. MAPbI_3_ is close to n-type with longer electron diffusion length but FAPbI_3_ is close to p-type with longer hole diffusion length, which guides design of perovskite layout. Understanding fundamentals of perovskite materials play important role in achieving high efficiency perovskite solar cell. In viewpoint of performance, high quality perovskite layer plays crucial role in overall photovoltaic parameters. Minimizing non-radiative recombination is one of methods to get high quality perovskite layer. High V_oc_ approaching band gap energy is expected if one can reach the theoretical V_oc_ deficit by engineering perovskite layer with highest internal PL quantum yield. A PCE of about 25 % can be realized using MAPbI_3_ and/or FAPbI_3_ when J_sc_, V_oc_ and FF reach 24 mA/cm^2^, 1.26 V (E_g_ = 1.6 V and V_oc_ deficit = 340 mV) and 0.83, respectively.
